# Implementation and utilization of Physical Examination Teaching Associate (PETA) programs: a scoping review

**DOI:** 10.1186/s41077-026-00416-z

**Published:** 2026-02-11

**Authors:** Holly Hopkins, Karen Lewis, Cathy M Smith, Marsha E. Yelen

**Affiliations:** 1 Eastern Michigan University School of Nursing, 311 Marshall Building, Ypsilanti, MI 48197 USA; 2 University of Texas at Tyler School of Medicine, 1100 South Beckham Avenue #3376, Tyler, TX 75701 USA; 3 Baycrest Academy for Research and Education at Baycrest Centre for Geriatric Care, 3560 Bathurst Street, Toronto, ON M6A 2X8 Canada; 4 Rush University Medical Center, 1620 W. Harrison St, Chicago, IL 60612 USA

**Keywords:** Physical examination teaching associate, Standardized patient, Standardized patient methodology, Simulated patient/participant, Professional patient, Patient instructor, Patient educator, Patient partner, Physical examination instruction, Musculoskeletal examination

## Abstract

**Background:**

Physical Examination Teaching Associates (PETAs) are individuals trained to instruct health professional learners regarding the technical and communication skills to conduct physical examinations. PETAs refer to their body to instruct and provide feedback in a supportive, non-threatening environment. Such programs are highly rated by learners and support learner outcomes, but implementation and utilization characteristics vary widely. This scoping review aims to review the literature addressing (1) the broad outcomes of PETA programs, (2) the utilization of PETA programs to instruct, and (3) the implementation of Domains of the ASPE SOBP and ASPE GTA/MUTA SOBP within PETA programs.

**Methods:**

PubMed, CINAHL, ERIC, PsycINFO, and Sociological Abstracts were searched to identify all publications addressing the administration of PETA programs and/or engagement of PETAs in instructional sessions. Studies were charted in tandem using an iterative process aligned with the PRISMA Extension for Scoping Reviews. The scoping review protocol was registered prospectively.

**Results:**

Thirty-four studies were identified. Most articles were from the United States and highlighted positive PETA program-level outcomes and learner outcomes. PETAs most frequently instructed individuals or groups of four second-year medical students learning the musculoskeletal examination. Half of the articles discussed engaging with PETAs with stable pre-existing pathology. Nearly all addressed some information related to the Domains of the ASPE SOBP and/or ASPE GTA/MUTA SOBP. Only two articles referred to the individual(s) engaging in the PETA role as a PETA.

**Conclusion:**

PETA programs have published positive outcomes for over 45 years. This scoping review reports on the available evidence highlighting the program structure and instructional patterns represented in the literature and the importance of consistent terminology and role description, alignment with publication standards, and implementation of the relevant ASPE SOBP.

**Registration:**

https://osf.io/rj5wp/.

**Supplementary Information:**

The online version contains supplementary material available at 10.1186/s41077-026-00416-z.

## Introduction

Guidelines for simulation-based healthcare training call for an integrated approach to teaching knowledge, skills, attitudes, and communication in healthcare education, including engaging with standardized/simulated patient/participants (SPs) for hands-on skills development [1]. Physical exams are vital to cultivating diagnostic accuracy and reducing unnecessary errors, which ultimately improves patient care [2, 3]. Despite their importance, physical exam teaching methods vary significantly by instructor type, curricular design, and framework used [2, 4, 5]. One method used in the United States, Canada, and Europe is the engagement of SPs, who assist in the formative and summative instruction of physical examinations [6, 7, 8], often as part of faculty-led instructional sessions. Similar to other instructional models, the effectiveness and standardization of SP-assisted physical exam training vary widely. Physical Examination Teaching Associates (PETAs), however, are SPs uniquely trained to instruct learners to perform hands-on physical examination skills. PETA instruction provides a standardized learning environment that increases cost efficiency and optimizes faculty time [2, 9–16].

According to the Healthcare Simulation Dictionary, PETAs are individuals “who are specifically trained to teach, assess, and provide feedback to learners about physical examination techniques. They can also address the communication skills needed” [17]. During a PETA session, learners perform a physical examination on the PETA, while the PETA simultaneously provides a formative assessment of their techniques through real-time feedback, drawing on both their physical experience of receiving the exam and their technical training on the necessary components of a physical examination. Beyond technical instruction, PETAs cultivate a supportive, non-threatening environment. This specialized focus ensures that learners prioritize safe and comfortable examination techniques, observe patient modesty, and maintain respectful communication throughout patient encounters.

A learner often receives minimal preparation (e.g., lecture, video) before a PETA instructional session and the PETA builds the learner’s confidence, competence, and knowledge through hands-on practice with immediate feedback [18]. Such formative feedback “involves clarification of goals, engaging the learner in reflection, self-assessment and dialog, providing instruction on how to modify the performance, and facilitating ‘opportunities to close the gap between the current and desired performance’ all while supporting the learner’s self-confidence” [19]. PETAs may have experience as SPs [20] or may be individuals with pathologic findings [15]. ​​ However, PETAs, like SPs, are distinct from patients. The term “patient” indicates an individual is receiving healthcare or treatment [21]. Patient involvement in healthcare providers’ education refers to patients drawing on their own lived experience to inform interactions with learners [22]. There are many reports of this approach in health professions education [22–26]. In contrast, PETAs are specifically trained as educators to systematically teach learners in a standardized manner.

PETA methodology was first reported in 1977 [27] alongside and based upon SP and Gynecologic Teaching Associate (GTA, female-bodied) methodologies [20, 28]. GTAs and Male Urogenital Teaching Associates (MUTAs, male-bodied) instruct pelvic, breast, urogenital, rectal, and/or prostate examinations [17, 29], while PETAs instruct other forms of physical examination, such as musculoskeletal, cardiovascular, and/or neurological examinations [17]. PETA programs have been documented in the United States, Canada, Australia, England, and Europe [9, 13, 27, 30, 31].

The interaction between a PETA and a learner is unique in that the learner examines the PETA’s body, and the PETA provides instruction and feedback based on that touch and observation. This is not a dynamic that many learners have experienced before encountering a PETA session. There is a power differential and distinct vulnerability for all participants, which increases the risk of psychological and physical safety for PETAs and learners. Fostering safety is therefore integral to every PETA session [18] as it is across broader SP, GTA/MUTA, and other simulation practices [29, 32–37]. These safety standards are grounded in the Association of SP Educators Standards of Best Practice (ASPE SOBP) [32] and the ASPE SOBPs for GTAs/MUTAs [29].

While SP methodology, defined in the ASPE SOBP, has evolved to become a broad umbrella that also encompasses PETA, GTA, and MUTA methodologies [17, 28, 29, 32], the current standards do not consider the unique aspects of PETA work. There is limited information and a lack of consensus about how to work with PETAs in a safe manner that supports positive learning outcomes. Furthermore, the implementation and utilization characteristics of PETA programs vary widely [38, 39]. The ASPE SOBP defines standards for SP programs with a focus on programs engaging SPs in role portrayal [32]. While PETAs are a type of SP, their role is more consistent with GTAs/MUTAs as they are both types of SPs who instruct physical examination skills. The ASPE GTA/MUTA SOBP [29] and ASPE SOBP [32] will be used in this paper to facilitate review of the existing literature. 

While scoping reviews and/or systematic reviews are emerging for aspects of patient involvement in education [24, 25] and SP methodology [40–44], there are no comprehensive reviews regarding PETA methodology. This scoping review was conducted to address this gap. We aimed to review the available evidence on the implementation and utilization of PETA programs in the education of health professional students. The following research questions were addressed:

- How are PETA programs utilized within the education of health professional learners?
- To what degree are the Domains within the ASPE SOBP [32] and/or ASPE GTA/MUTA SOBP [29] used to describe the implementation of PETA programs?
- What broad outcomes do current PETA publications address?

## Methods

The protocol was drafted using the Preferred Reporting Items for Systematic Reviews and Meta-Analysis Protocols (PRISMA-P) [45]. The protocol was registered prospectively with the Open Science Framework [46]. The scoping review was an iterative process aligned with the Arksey and O’Mally methodological framework [47] and the PRISMA Extension for Scoping Reviews [48].

The authors of this manuscript were the researchers. The authors have significant experience working with PETAs (KL), SPs (KL, CS, MY), and GTAs/MUTAs (HH) in the United States and Canada. Our backgrounds additionally include nursing (HH, MY), education (KL), and performance (CS). We began this scoping review as members of the ASPE Standards of Practice Committee, which creates SOBP for SP Educators. Individual experiences informed discussion at each stage of the scoping review process, which proved valuable, especially in checking assumptions and bias while calibrating our understanding. We met frequently in pairs and as a group to discuss, reflect, and come to consensus on our findings.

### Information sources

**Fig. 1:**
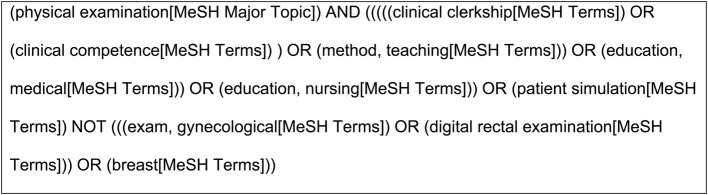
PubMed search strategy

A pilot search was conducted in July 2020 using the key words “physical teaching associate” and “medical education”. Seven hundred and twenty-five articles, all from medicine and nursing programs, were identified and screened for relevance. Titles, keywords, and index terms from these articles were analysed for relevance to further enhance the keyword search. PubMed, CINAHL, ERIC, PsycINFO, and Sociological Abstracts were searched on March 18, 2021. PubMed, CINAHL, and ERIC address healthcare, healthcare education, and education. A prior review of the related GTA/MUTA methodology [43] demonstrated citations of GTA/MUTA methodology in sociological and psychological databases, thus the inclusion of PsycINFO and Sociological Abstracts. The search strategy for PubMed is available in Fig. 1. Reference lists of included articles were hand-searched to identify additional relevant literature. Authors were not contacted for further information regarding their publication(s).

### Eligibility criteria

Articles were included if PETA(s) were engaged in instructional sessions with learners to conduct physical examinations and/or if they mentioned the administration of a PETA program. Articles were included if they were published before our search (March 18, 2021) with full-text available in the English language. We lacked the resources necessary to perform translations.

Exclusion criteria were developed through group consensus and primarily included articles not addressing PETA methodology and Letters to the Editor, as seen in Table 1. Extensive discussion occurred regarding the definition of PETA methodology and its application in the literature resulting in opportunities to refine the definition of PETA, as noted in the Discussion.

**Table 1:** Exclusion criteria

Letter to the Editor	
Books	
Does not address PETA methodology	Articles where a human was functioning as a model with a healthcare professional providing all instruction
	Articles addressing peer instruction (i.e. a medical student teaching a medical student, even if referring to their own body for instructional purposes)
	Articles where a PETA did not provide any initial instruction, only follow-up corrective feedback after a physical examination was performed
	Articles addressing summative assessment of focused physical examination skills that are not conducted by PETA
	Articles where individuals with pathology provide instruction on their unique pathology (i.e. this *my unique presentation of arthritis* versus this is how you conduct a musculoskeletal assessment)
	Articles addressing instruction of only breast, pelvic, urogenital, rectal, and/or prostate examination

### Article selection

Covidence (https://www.covidence.org/) was used to manage documents, facilitate screening, and conduct data extraction.

Researchers independently evaluated each title and/or abstract. If the title and/or abstract addressed PETA methodology or physical examination instruction without clearly excluding PETA methodology it moved forward. Researchers reviewed the first thirty titles and abstracts in duplicate to ensure the team’s understanding of inclusion/exclusion criteria.

All articles that met title/abstract inclusion criteria were reviewed in full to determine whether inclusion criteria were met. Two randomized reviewers reviewed the first twenty full-text articles to evaluate consistency among the team.

Article selection followed an iterative process, with ongoing refinement of exclusion criteria. Uncertainties were resolved through discussion and group consensus. Once consensus was identified, the remaining articles were screened independently.

Reference lists of included articles were hand-searched.

### Data charting

The data extraction form was developed iteratively with extensive group discussion. Once consensus was reached, all included articles were charted in Covidence in duplicate by pairs (HH and KL, CS and MY) and results were discussed by the pair to reach consensus. For each included article, we charted items as listed in Table 2. Methodological quality and bias were not assessed. The Online Supplementary Materials hosts tables of the charted data. The data is divided into four tables: publication characteristics; broad outcomes; PETA program utilization patterns and structure; and, implementation.

**Table 2:** Data charting items

Publication characteristics	Country Study design Terminology used to describe PETAs
Broad outcomes	Self-assessment completed Learner perception of program Whether and how learner assessment took place after the instructional session PETA program-level outcomes PETA experiences Overall assessment of program Whether outcomes were positive, negative, or mixed (defined as an improvement in variables assessed in the article)
PETA program utilization patterns and structure	Learner type and number Curricular timing Learner preparation Session length Type of physical examination instructed Length of PETA training Type of instruction (independent vs. paired) PETA wage How learners were taught before the PETA program Size of PETA program PETAs with pre-existing pathology
Program implementation	Whether the Domains of the ASPE SOBP (32) and ASPE GTA/MUTA SOBP (29) were addressed

### Collating, summarizing and reporting the results

The analysis of key characteristics of the data was summarized and reported in the results below.

## Results

**Fig. 2:**
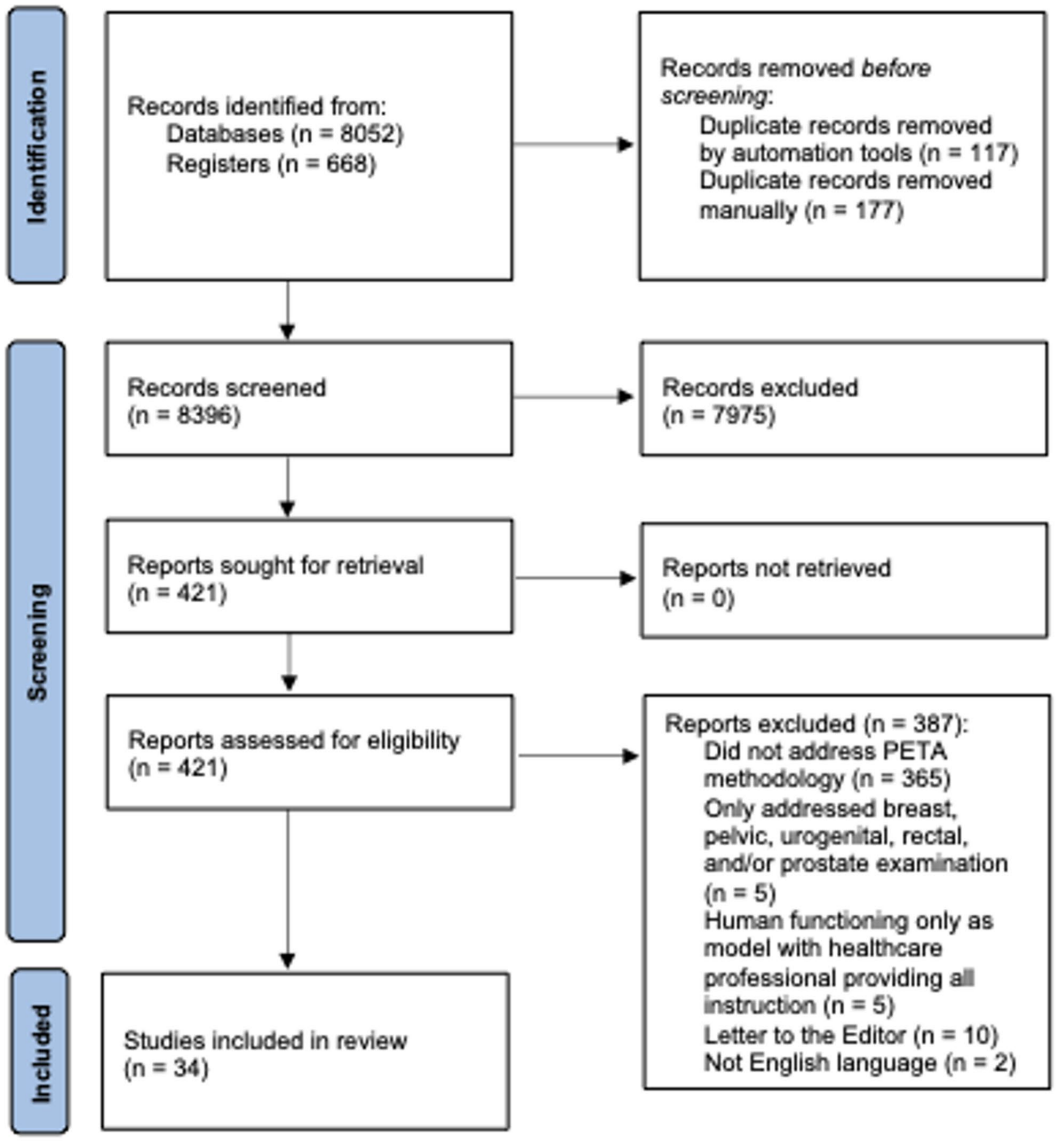
PRISMA flow diagram

Of the 8,720 articles identified in March 18 2021, 8,396 were original articles screened at the title/abstract level for inclusion. Those articles that met the inclusion criteria or did not have an abstract progressed to full-text screening. 421 full-text articles were assessed with 34 articles meeting inclusion criteria (Fig. 2 and Online Supplemental Materials, Included References).

### Publication characteristics

**Fig. 3:**
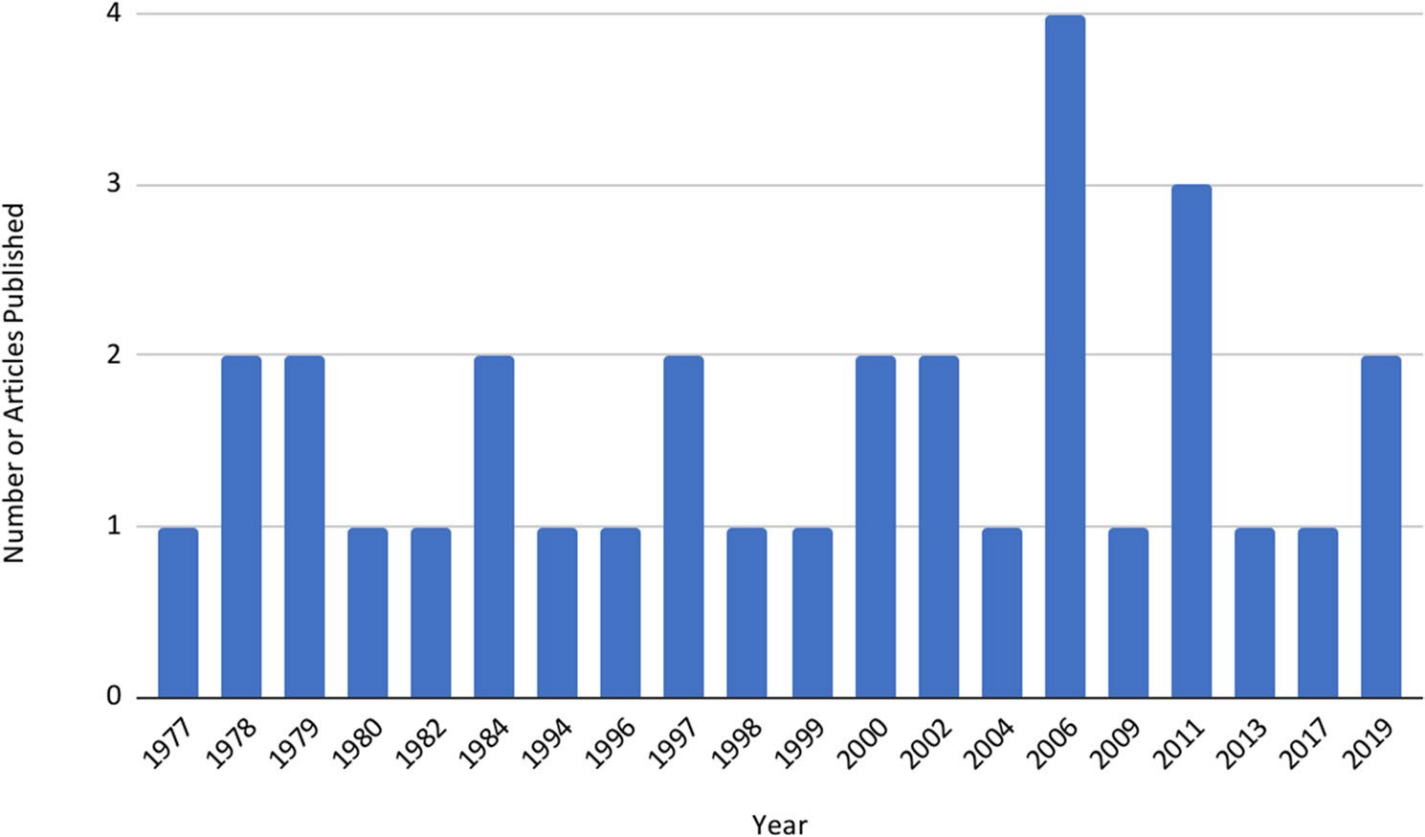
PETA publication timeline

**Fig. 4:**
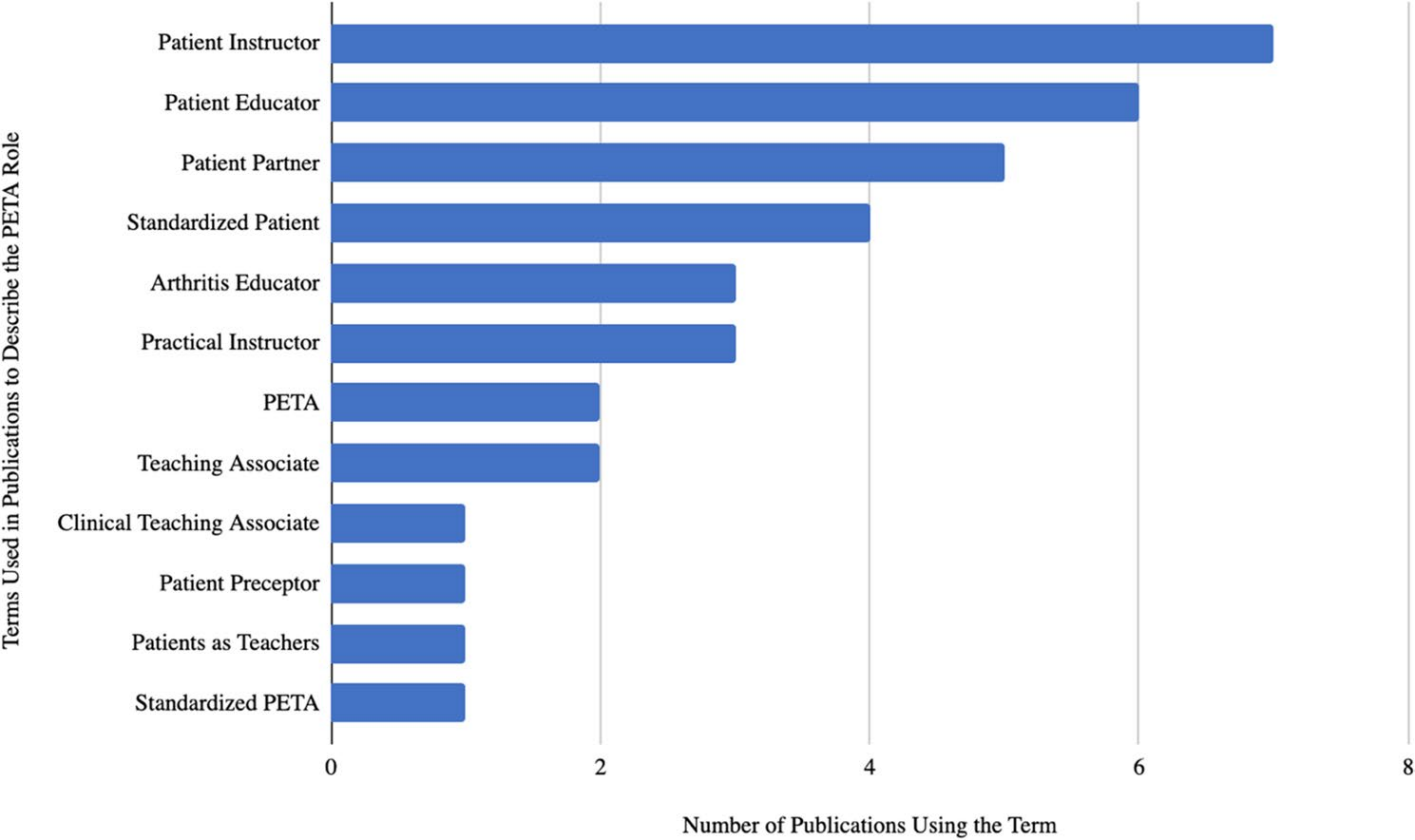
Terminology used when discussing PETAs

59% of the articles were from the United States (*n* = 20) followed by Canada (*n* = 6), England (*n* = 4), Australia (*n* = 3), and Germany (*n* = 2). As demonstrated in Fig. 3, four publications were released in 2006, but most other years revealed one or two publications. Most studies were quantitative (*n* = 22) and reported overall positive outcomes (*n* = 29), although outcomes were defined in different ways. Variable terminology was used to discuss PETAs, with Patient Instructor (*n* = 7) and Patient Educator (*n* = 7) being the most common (see Fig. 4 and Online Supplementary Materials, Table 1, Publication Characteristics of PETA Studies).

### Overall outcomes

PETA program-level outcomes were addressed in 28 articles, and the experiences of PETAs were addressed in 10. Learners reported on their perception of the PETA program (*n* = 18) and self-assessed their comfort (*n* = 5) and competence (*n* = 9). Twenty-four articles reported on the assessment of learners following initial PETA instruction; where reported, this external assessment was completed by PETAs (*n* = 12), faculty (*n* = 5), or other examiners (*n* = 3). None of the articles that discussed learner self-evaluation or external assessment provided specific information about the assessment tools (see Online Supplementary Materials, Table 2, Broad Outcomes of PETA Studies).

### Program structure and instructional patterns

**Fig. 5:**
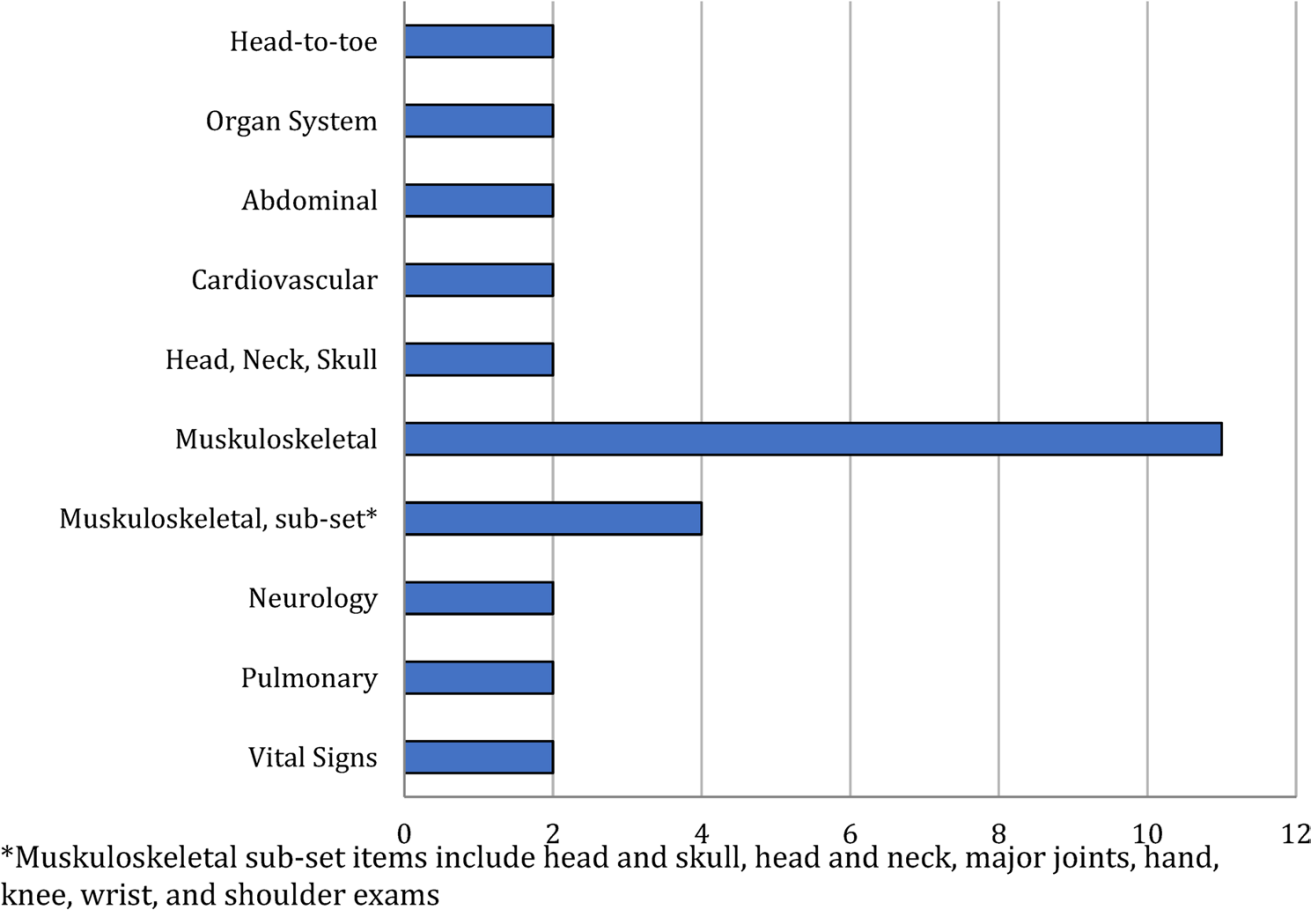
Physical examinations instructed

Medical students most frequently worked with PETAs (*n* = 29; physicians, *n* = 5; nurses, *n* = 1; not addressed, *n* = 3). PETAs mostly worked with groups of one (*n* = 9) or four (*n* = 7) although groups of two, three, six, seven, eight, and ten or more (*n* = 1 each; 22 total reported, mean = 3.3, SD = 2.59) were also represented. Depending on the country and the length of the training program, learners were most commonly in their 2nd year of schooling (15/24) followed by 1st and 3rd (*n* = 7 each) then 4th and 5th (*n* = 1 each). As noted in Fig. 5, most studies discussed instruction of the musculoskeletal examination (*n* = 11); five studies lacked sufficient detail to characterize the examination type. Learner preparation before the instructional session was addressed in 22 articles and varied significantly. See Online Supplementary Materials, Table 3, Program Structure and Utilization Patterns for further details.

Programs described having an average of eight PETAs (*n* = 15, min = 2, max = 20). Seventeen programs reported including PETAs based on pre-existing pathology, most commonly arthritis. Of the twenty-one articles that reported on length of PETA training, the range was 3 to 240 hours although specifics were not thoroughly reported so statistical analysis is not possible (see Online Supplementary Materials, Table 3, PETA Program Structure and Utilization Patterns). Only one of 15 PETA programs reported working in pairs with another PETA, the remaining 14 worked independently. PETA wages were reported in 11 articles, but comparison of compensation is not possible because of variables such as location, year the article was published, and the PETA’s role responsibility. (see Online Supplementary Materials, Table 3, PETA Program Structure and Utilization Patterns).

Before the implementation of the PETA program, most learners were taught by faculty (10/13 reporting) with clinical patients (*n* = 6), peers (*n* = 3), or SPs (*n* = 2) receiving the examination (*n* = 9).

### ASPE SOBP implementation

**Fig. 6:**
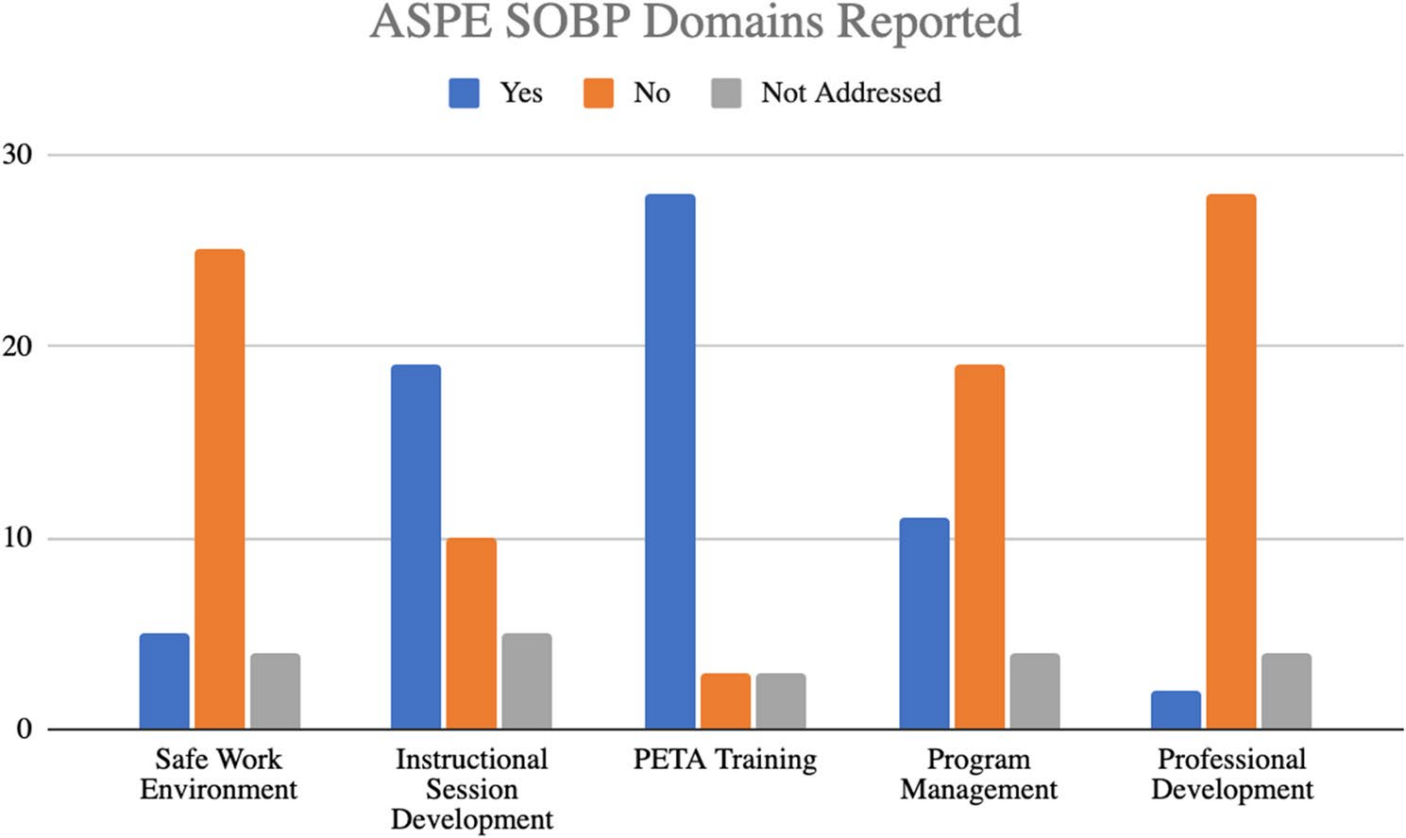
ASPE SOBP domains reported

Thirty-one articles included some information relevant to the Domains of the ASPE SOBP [32] and/or the ASPE GTA/MUTA SOBP [29]. As noted in Fig. 6, the Training Domain (*n* = 28) was most commonly addressed, followed by the Instructional Session Development Domain (*n* = 19). Variance in the Not Addressed columns reflects the variance in article methodology and aims (see Online Supplementary Materials, 4, Implementation of PETA Programs).

## Discussion

This review provides a comprehensive overview of the published evidence on the implementation and utilization of PETA programs in the education of medical and nursing students. Overall, PETA programs have positive outcomes [2, 9–11, 14–16, 20, 26, 27, 38, 39, 49–56]. They are highly rated by learners [9, 10, 12, 15, 20, 27, 30, 31, 38, 39, 50, 53, 55–60] and support learners’ self-assessed comfort [12, 38, 39, 53, 59] and competence [9, 12, 31, 38, 39, 54, 57, 59, 61]. External assessment of the learner after a PETA instructional session was also overall positive [2, 9, 11–13, 16, 20, 27, 31, 39, 49–51, 53–55, 57, 58, 61, 62]. While many program characteristics are reported on, there is limited evidence for other features, such as wage [8–13, 15, 16, 38, 50, 59] and PETA training [9–16, 20, 26, 27, 30, 31, 38, 39, 49–54, 56–60, 62, 63]. We reported on 34 articles that clearly met inclusion criteria while many other articles, particularly those about SP roles, helped inform our exclusion criteria and the proposed definition of PETA methodology. The results of this scoping review inform our recommendations for future areas for research, as discussed below and listed in Table 3.

**Table 3:** Future research areas

Pedagogical implementation strategies
· Outcomes and objectives of the instructional session
· Time in the curriculum
· Time per learner
· Time per system or examination type
· Description of interaction between the PETA and the learner
· Description of PETA training
· Description of learner preparation
· Identification of variables that enhance outcomes
· Incorporation of trauma-informed teaching practices (e.g.,the PETA asking the learner if they may touch their hand to adust their palpation technique)
Humanization and Ethics
· Demographics of PETAs
· PETA reports of the risks and benefits of their role
· PETA reflection on pedagogical implementation strategies
· PETA reflection on policies and protocols to enhance their safety and that of their learners
Methodological Comparison
· Compare and contrast PETA, SP, GTA, and MUTA program structure
· Compare and contrast PETA, GTA, and MUTA pedagogical implementation strategies

### Terminology and role description

The approved terminology for this role is “Physical Examination Teaching Associate” or “PETA”. That name was used in only two of the articles reviewed. This creates challenges for literature reviews and effective communication with colleagues. For example, Davidson [64] discussed “PETAs” who oversee peer exams and provide feedback to learners who practice the examination techniques on their peers. While this technique offered significant cost savings to the program, it is not an accurate description of PETA methodology. As early as 1993, Barrows [65] offered the following definitions: “Practical instructors have been trained to teach the pelvic or genitorectal examination as it is being performed on them by the student. Patient instructors, a contribution of Paula Stillman, are patients who have been carefully educated about their own illnesses and how they should be evaluated on history and physical”. However, these definitions do not appear to align with application in the literature either.

We propose the following revision to the current PETA [17] definition to more thoroughly describe the role: Physical Examination Teaching Associates (PETAs or PTAs) are individual(s) (e.g., SP, community member) who are specifically trained to teach physical examination techniques in a standardized manner with their own body. Within a formative context, learners perform the physical examination techniques on the PETA. The PETA simultaneously provides real-time feedback to the learner based on their experience receiving the exam, which may be incorporated into subsequent attempts within the instructional session. They also teach learners the communication skills to conduct the exam with dignity and respect. PETAs work to cultivate a supportive, non-threatening learning environment. Some PETAs may have stable pathology present, but they teach more broadly about physical examination techniques beyond their own unique presentation.

Clear role descriptions for PETAs additionally support program goals and the physical and psychological safety of PETAs and learners [32]. Several excluded articles discussed SP physical examination instruction but lacked sufficient detail to identify whether the SPs were engaging in PETA or SP methodology [7, 66]. There may be more literature addressing PETA methodology than we located given the inability to clearly discern roles. Pairing standardized terminology with a brief description of the interaction between the PETA and the learner will ensure clear communication and advance the professionalization of PETA work. The importance of precise and respectful language to describe roles in simulation [43, 44, 67–69] and an underreporting of the simulation methodology [41, 44, 70, 71] is echoed in broader simulation practices literature.

### Depth of publication

A recent scoping review [43] addressing GTA/MUTA methodology yielded 101 articles. Despite developing at similar times, there are 66% fewer PETA publications when compared to the total number of GTA/MUTA publications. We expected a similar number of publications and wonder if this is due to inconsistent terminology and discussion of the role or some other reason. There are many possible explanations for this disparity, including but not limited to social stigmatization of genitalia increasing GTA/MUTA publications, lack of regard for other portions of the anatomy, limited evidence on physical examination instruction overall, misuse of terminology leading to missed publications, or an assumption that current SP research already addresses PETA methodology. Future research should explore the similarities and differences between PETA and GTA/MUTA programs and the overlap between PETA and SP programs to help further develop and professionalize the PETA role (Table 3).

### Program structure and instructional patterns

Nearly all publications on PETA methodology highlighted positive outcomes, broadly defined. Effective pedagogy is of utmost importance in health professional education and physical examination skills form the foundation of a healthcare provider’s skillset. Descriptions of what aspects of physical examination instruction result in positive outcomes have not been fully identified [2]. For example, timing within the curriculum, the amount of time per learner, learner preparation, number of exams per PETA per day, and PETA training may all impact learner outcomes, but these variables have not been fully explored and/or are underreported. This conclusion has also been reached in many other simulation reviews [1, 41, 44, 70, 71]. The number of examinations a PETA instructs each session or each day may impact both the learner’s experience and the PETA’s perceptions or comfort [52]. Studies that identify the variables that make PETA programs as safe and effective as possible are critical to ensure well-prepared health professional learners. Aligning with SP publication recommendations [71], reporting guidelines related to broader healthcare simulation research such as the CONSORT statement [70], and findings reported in previous research focused on SP methodology [41, 42, 44, 72, 73] will enhance the rigor of PETA publications. Such rigor will help PETA programs facilitate safety and effective pedagogy.

Many programs trained individuals with specific and stable pathological findings to instruct learners on the physical examination techniques relevant to their pathology [9, 10, 14, 15, 26, 27, 31, 39, 51, 52, 54, 55, 57, 60, 61, 63, 64]. The results of these programs are very positive. Although there was some analysis of the PETA’s experience, it is an insufficient exploration of the human experience in this role. PETAs are humans and may experience the same potential harms as patients seeking healthcare. It is our ethical and moral imperative to support their safety and well-being in this work through the incorporation of trauma-informed policies and protocols that facilitate safe and effective instruction [74]. Utilizing SOBP tailored specifically to PETA programs (e.g., the forthcoming ASPE PETA SOBP [18]) will additionally support safety of PETAs and efficacy of PETA programs.

### Implementation of ASPE SOBP

The overwhelming majority of the articles were written before the ASPE SOBP [32] and ASPE GTA/MUTA SOBP [29] were published. The ASPE SOBP is applicable because PETAs are a subset of SPs and therefore PETA programs should be administered by SP Educators. The ASPE GTA/MUTA SOBP is applicable because GTAs/MUTAs and PETAs have similar methodologies. The two SOBP are very similar but hold important differences related to case portrayal and instructional session delivery. The ASPE PETA SOBP [18] is forthcoming and should be consulted for PETA program development and related publications. This scoping review will inform the ASPE PETA SOBP, which will support SP Educators who work with PETA programs and simulation educators in general to understand the complexities of SP and PETA work.

### Limitations

Due to variances in terminology, indexing, and reporting of engagement with PETA programs, some publications may have been omitted. Books discuss PETA methodology [75] but were omitted from this review. Relevant articles may have been published since this search was completed. Seventeen articles were published since 2000, which is an average of 0.7 articles per year. We anticipate that the gap between completing the search and publishing may result in no more than two or three missed articles.

Data charting may be incomplete due to variance in how data was reported in publishing. For example, some articles discuss engaging SPs in physical examination instruction [66]; while one may infer that the SPs are or may be working in a PETA role, if that was not explicitly stated the article was excluded. This again highlights the need for standardized terminology. Consistent with scoping review methodology, we did not assess the articles for quality or bias, indicating another possible limitation of this review.

This review does not necessarily reflect the scope of PETA programs that exist globally; many programs may not engage in research and others may have published with incomplete details or in another language. Only two articles were excluded from the search because they were not published in English, so our inability to translate does not appear to significantly impact our results.

While we followed an explicit, registered protocol, researchers also may impart their own bias. We mitigated this by following the protocol and charting in tandem. We did not engage in a consultation exercise with relevant groups, however for the accompanying ASPE PETA SOBPs [18], we did consult with numerous relevant parties using the Delphi process.

## Conclusion

PETA programs have reported positive outcomes for over 45 years. This scoping review does not provide recommendations for program implementation or utilization but does report on the available evidence for PETA programs and propose future research questions and actions. Utilizing consistent terminology and descriptions of the PETA/learner interaction in publications that are aligned with current simulation-reporting guidelines [70, 71], ethical guidelines, and the ASPE SOBP [29, 32] (including the forthcoming ASPE PETA SOBP [18]) will support the advancement of this important instructional method.

## Supplementary Information

Supplementary Material 1. Table 1. Publication characteristics of PETA Studies. Table of country where the study took place, study design, and terminology used to describe PETA.

Supplementary Material 2. Table 2. Broad Outcomes of PETA Studies. Table of learner self-assessment and type of tool, learner perception of PETA program, assessment of learner after PETA instruction, who completed assessment of the learner and what tool, PETA program level outcomes, experience of PETAs, overall assessment of outcomes.

Supplementary Material 3. Table 3. PETA program structure and utilization patterns. Table of learner type, number of learners, timing of PETA instructional session within curriculum, learner preparation, session length, physical examination(s) instructed, length of initial training for PETA, independent vs paired PETA instruction, wage, who instructed learners and received examination(s) before PETA program, number of PETAs in program, whether PETAs were included because of pre-existing pathology.

Supplementary Material 4. Table 4. Implementation of PETA Programs. Table of whether details were provided about the ASPE SOBP Domains (safe work environment, instructional session development, PETA training, program management, professional development).

Supplementary Material 5. Studies meeting inclusion criteria.

## Data Availability

The datasets supporting the conclusions of this article are included within the article and its additional files.

## Abbreviations

ASPE: Association of SP Educators
GTA: Gynecological Teaching Associate
MUTA: Male Urogenital Teaching Associate
PETA: Physical Examination Teaching Associate
SOBP: Standards of Best Practice
SP: Standardized or simulated patient or participant
