# Supplementary material for: Implementation and utilization of Physical Examination Teaching Associate (PETA) programs: a scoping review
Source: Adv Simul (Lond). 2026 Feb 11;11:13. doi: 10.1186/s41077-026-00416-z (PMC12922200; doi:10.1186/s41077-026-00416-z)
Supplement: Supplementary file 1 — Supplementary Material 1. Table 1. Publication characteristics of PETA Studies. Table of country where the study took place, study design, and terminology used to describe PETA. [file 41077_2026_416_MOESM1_ESM.pdf]

Implementation and Utilization of Physical Examination Teaching Associate (PETA) Programs: A Scoping Review

Online Supplementary Materials

Table 1. Publication Characteristics of PETA Studies

Author(s)	Year of Publication	Country Where Study Took Place	Study Design					Terminology to Describe PETA
			Qualitative	Quantitative	Mixed Methods	Literature Review	Other	
Aamodt, Virtue, Dobbie	2006	United States		x				Physical examination teaching associate
Allen, Miller, Ratner, Santilli	2011	United States		x				Patient educator
Barley, Fisher, Dwinnell, White	2006	United States		x				Standardized physical examination teaching associate
Barnes, Albanese, Schroeder, Reiter	1978	United States			x			Patient preceptor
Bell, Badley, Glazier, Poldre	1997	Canada			x			Patient educator, patient partner
Branch, Graves, Hanczyc, Lipsky	1999	United States		x				Arthritis educator
Branch, Lipsky	1998	United States		x				Arthritis educator, patient instructor
Danielson, Venugopal, Mefford, Clarke	2019	United States				x		Physical examination teaching associate
Errichetti, Gimpel, Boulet	2002	United States		x				Standardized patient
Frazer, Miller	1977	United States					x	Practical instructor
Gall, Meredith, Stillman, Rutala, Gooden, Boyer, Riggs.	1984	United States					x	Patient instructor
Gruppen, Branch, Laing	1996	United States		x				Arthritis educator
Haq, Fuller, Dacre	2006	England			x			Patient partner
Hasle, Anderson, Szerlip	1994	United States		x				Standardized patient
Hendry, Schrieber, Bryce	1999	Australia			x			Patient partner
Hoefer, Sterz, Bender, Stefanescu, Theis, Walcher, Sader, Ruesseler	2017	Germany		x				Teaching associate
Howley, Gliva-McConvey, Thornton	2009	Other: United States and Canada		x				Standardized patient
Humphrey-Murto, Smith, Touchie, Wood	2004	Canada		x				Patient educator
Laguna, Stillman	1978	United States		x				Practical instructor
Martineau, Mamede, St-Onge, Rikers, Schmidt	2013	Canada		x				Patient instructor
Oswald, Bell, Wiseman, Snell	2011	Canada		x				Patient educator
Oswald, Wiseman, Bell, Snell	2011	Canada	x					Patient educator
Parle, Ross, Coffey	2012	England					x	Clinical teaching associate
Raj, Badcock, Brown, Deighton, O'Reilly	2006	England		x				Patient educator
Riggs, Gall, Meredith, Boyer, Gooden	1982	United States			x			Patient instructor
Sachdeva, Wolfson, Blair, Gillum, Gracely, Friedman	1997	United States		x				Standardized patient
Schrieber, Hendry, Hunter	2000	Australia		x				Patient partner
Smith, Henry-Edwards, Shanahan, Ahern	2000	Australia		x				Patient partner

Implementation and Utilization of Physical Examination Teaching Associate (PETA) Programs: A Scoping Review

Online Supplementary Materials

Table 1. Publication Characteristics of PETA Studies

Author(s)	Year of Publication	Country Where Study Took Place	Study Design					Terminology to Describe PETA
			Qualitative	Quantitative	Mixed Methods	Literature Review	Other	
Stillman	1984	United States					x	Patient instructor
Stillman, Levinson, Ruggill, Sabers	1979	United States		x				Practical instructor
Stillman, Ruggill, Rutala, Sabers	1980	United States		x				Patient instructor
Stillman, Ruggill, Rutala, Sabers	1979	United States		x				Patient instructor
Wykurz, Kelly	2002	England				x		Patients as teachers
Zabel, Sterz, Hoefer, Stefanescu, Lehmann, Sakmen, Marzi, Ruesseler	2019	Germany		x				Teaching associate
