# Supplementary material for: Implementation and utilization of Physical Examination Teaching Associate (PETA) programs: a scoping review
Source: Adv Simul (Lond). 2026 Feb 11;11:13. doi: 10.1186/s41077-026-00416-z (PMC12922200; doi:10.1186/s41077-026-00416-z)
Supplement: Supplementary file 2 — Supplementary Material 2. Table 2. Broad Outcomes of PETA Studies. Table of learner self-assessment and type of tool, learner perception of PETA program, assessment of learner after PETA instruction, who completed assessment of the learner and what tool, PETA program level outcomes, experience of PETAs, overall assessment of outcomes. [file 41077_2026_416_MOESM2_ESM.pdf]

Implementation and Utilization of Physical Examination Teaching Associate (PETA) Programs: A Scoping Review

Online Supplementary Materials

Table 2. Broad Outcomes of PETA Studies

Author(s)	Year of Publication	If learners completed a self-assessment, what did that cover?				What tool(s) did the learner use for self-assessment?		Did learners report on their overall perception of the PETA program?			Did assessment of learner take place after PETA instruction?			Who completed the external assessment of the learner?				What tool(s) did the external evaluator use?		Were PETA program level outcomes addressed?			Was the experience of the PETAs addressed?			Overall assessment of outcomes
		Comfort	Competence	Other	Not Applicable	Institution-Specific Tool	Not Addressed	Yes	No	Not Addressed	Yes	No	Not Addressed	PETA	Faculty	Other	Not Addressed	Institution-Specific Tool	Not Addressed	Yes	No	Not Applicable	Yes	No	Not Applicable	
Aamodt, Virtue, Dobbie	2006	x	x			x		x				x					x		x	x				x		Positive
Allen, Miller, Ratner, Santilli	2011				x		x			x	x			x				x		x				x		Positive
Barley, Fisher, Dwinnell, White	2006				x		x			x	x			x				x		x				x		Positive
Barnes, Albanese, Schroeder, Reiter	1978				x		x	x			x				x			x		x			x			Positive
Bell, Badley, Glazier, Poldre	1997				x		x	x			x						x		x	x				x		Mixed
Branch, Graves, Hanczyc, Lipsky	1999				x		x			x	x			x				x		x				x		Positive
Branch, Lipsky	1998	x	x	x		x		x			x			x				x		x			x			Positive
Danielson, Venugopal, Mefford, Clarke	2019				x		x			x	x						x		x	x				x		Positive
Errichetti, Gimpel, Boulet	2002				x		x			x			x				x		x		x			x		Positive
Frazer, Miller	1977				x		x	x			x				x			x		x				x		Positive
Gall, Meredith, Stillman, Rutala, Gooden, Boyer, Riggs	1984				x		x	x					x				x		x	x			x			Positive
Gruppen, Branch, Laing	1996		x	x		x		x			x						x		x	x			x			Positive
Haq, Fuller, Dacre	2006	x	x	x		x		x			x						x	x		x			x			Mixed
Hasle, Anderson, Szerlip	1994	x	x			x		x			x						x	x		x				x		Positive
Hendry, Schrieber, Bryce	1999		x				x	x			x			x					x	x			x			Positive
Hoefer, Sterz, Bender, Stefanescu, Theis, Walcher, Sader, Ruesseler	2017				x		x			x	x				x			x		x				x		Positive

Implementation and Utilization of Physical Examination Teaching Associate (PETA) Programs: A Scoping Review

Online Supplementary Materials

Table 2. Broad Outcomes of PETA Studies

Author(s)	Year of Publication	If learners completed a self-assessment, what did that cover?				What tool(s) did the learner use for self-assessment?		Did learners report on their overall perception of the PETA program?			Did assessment of learner take place after PETA instruction?			Who completed the external assessment of the learner?				What tool(s) did the external evaluator use?		Were PETA program level outcomes addressed?			Was the experience of the PETAs addressed?			Overall assessment of outcomes
		Comfort	Competence	Other	Not Applicable	Institution-Specific Tool	Not Addressed	Yes	No	Not Addressed	Yes	No	Not Addressed	PETA	Faculty	Other	Not Addressed	Institution-Specific Tool	Not Addressed	Yes	No	Not Applicable	Yes	No	Not Applicable	
Howley, Gliva-McConvey, Thornton	2009				x		x			x			x				x		x		x			x		Other
Humphrey-Murto, Smith, Touchie, Wood	2004				x	x		x			x				x			x			x			x		Negative
Laguna, Stillman	1978				x		x	x			x			x				x		x				x		Positive
Martineau, Mamede, St-Onge, Rikers, Schmidt	2013				x		x			x	x			x				x			x			x		Positive
Oswald, Bell, Wiseman, Snell	2011		x			x			x		x			x				x		x				x		Positive
Oswald, Wiseman, Bell, Snell	2011				x		x			x			x				x		x	x				x		Positive
Parle, Ross, Coffey	2012				x		x			x			x				x		x		x			x		Other
Raj, Badcock, Brown, Deighton, O'Reilly	2006		x	x		x		x			x				x				x	x			x			Positive
Riggs, Gall, Meredith, Boyer, Gooden	1982				x		x			x			x				x		x	x			x			Positive
Sachdeva, Wolfson, Blair, Gillum, Gracely, Friedman	1997	x				x		x			x			x				x		x				x		Positive
Schrieber, Hendry, Hunter	2000		x			x				x	x			x		x		x		x				x		Positive
Smith, Henry-Edwards, Shanahan, Ahern	2000				x	x		x			x					x		x		x					x	Positive
Stillman	1984				x		x			x			x				x		x	x			x			Positive
Stillman, Levinson, Ruggill, Sabers	1979				x		x	x				x					x		x	x					x	Positive
Stillman, Ruggill, Rutala, Sabers	1980				x		x	x			x			x				x		x				x		Positive
Stillman, Ruggill, Rutala, Sabers	1979				x		x	x			x			x				x		x				x		Positive

Implementation and Utilization of Physical Examination Teaching Associate (PETA) Programs: A Scoping Review

Online Supplementary Materials

Table 2. Broad Outcomes of PETA Studies

Author(s)	Year of Publication	If learners completed a self-assessment, what did that cover?				What tool(s) did the learner use for self-assessment?		Did learners report on their overall perception of the PETA program?			Did assessment of learner take place after PETA instruction?			Who completed the external assessment of the learner?				What tool(s) did the external evaluator use?		Were PETA program level outcomes addressed?			Was the experience of the PETAs addressed?			Overall assessment of outcomes
		Comfort	Competence	Other	Not Applicable	Institution-Specific Tool	Not Addressed	Yes	No	Not Addressed	Yes	No	Not Addressed	PETA	Faculty	Other	Not Addressed	Institution-Specific Tool	Not Addressed	Yes	No	Not Applicable	Yes	No	Not Applicable	
Wykurz, Kelly	2002				x		x			x			x				x		x			x	x			Positive
Zabel, Sterz, Hoefer, Stefanescu, Lehmann, Sakmen, Marzi, Ruesseler	2019				x		x			x	x					x		x		x				x		Positive
