# Supplementary material for: Implementation and utilization of Physical Examination Teaching Associate (PETA) programs: a scoping review
Source: Adv Simul (Lond). 2026 Feb 11;11:13. doi: 10.1186/s41077-026-00416-z (PMC12922200; doi:10.1186/s41077-026-00416-z)
Supplement: Supplementary file 3 — Supplementary Material 3. Table 3. PETA program structure and utilization patterns. Table of learner type, number of learners, timing of PETA instructional session within curriculum, learner preparation, session length, physical examination(s) instructed, length of initial training for PETA, independent vs paired PETA instruction, wage, who instructed learners and received examination(s) before PETA program, number of PETAs in program, whether PETAs were included because of pre-existing pathology. [file 41077_2026_416_MOESM3_ESM.pdf]

Implementation and Utilization of Physical Examination Teaching Associate (PETA) Programs: A Scoping Review

Online Supplementary Materials

Table 3. PETA Program Structure and Utilization Patterns

Author(s)	Year of Publication	Learner type				Number of learners in each session											Timing of initial PETA instructional session within curriculum						How is learner preparation prior to instructional session with PETA described?		Session length		Type of physical examination(s) instructed		Length of initial training for PETA		Were PETAs independent or paired for initial instructional session(s)?			Wage		Prior to PETA involvement, who instructed the students?				Prior to PETA involvement, who/what received the exams?				Number of PETAs in program		Were PETAs included because of pre-existing pathology?		
		Medical Student(s)	Nurse Practitioner Student(s)	Physician(s)	Not Addressed	1	2	3	4	5	6	7	8	9	10 or more	Not Addressed	1st year	2nd year	3rd year	4th year	5th year	Not Addressed	Description Provided	Not Addressed	Time Provided	Not Addressed	Specific Examination(s)	Not Specified	Time Provided	Not Addressed	Independent	Paired with Peer (PETA)	Not Addressed	Wage Provided	Not Addressed	Faculty	Senior Medical Students	Other	Not Addressed	Clinic Patients	Peers	SP	Not Addressed	Number Provided	Not Addressed	Yes	No	Not Addressed
Aamodt, Virtue, Dobbie	2006	x							x								x						x		7x120 min		organ system		3 hours per system		x			$15-20/hour		x					x			10			x	
Allen, Miller, Ratner, Santilli	2011	x							x									x					x		6x110 min		head-to-toe		12 hours				x	$18/hour		x						x		18			x	
Barley, Fisher, Dwinnell, White	2006	x				x											x							x	390 min		musculoskeletal, cardiovascular, pulmonary, abdominal, vital signs		6 hours per system		x				x				x				x		x			x
Barnes, Albanese, Schroeder, Reiter	1978	x							x									x					x		10x120 min			x	3 hours				x	$60 in text books					x				x	16			x	
Bell, Badley, Glazier, Poldre	1997	x		x		x																x	x		90 min		joint			x	x				x				x				x		x			x
Branch, Graves, Hanczyc, Lipsky	1999			x		x												x	x				x			x	musculoskeletal, arthritis		25 hours		x				x			x		x					x	x		
Branch, Lipsky	1998	x				x												x					x		90 min		musculoskeletal		6 weeks				x		x				x				x		x	x		
Danielson, Venugopal, Mefford, Clarke	2019	x														x	x	x						x		x		x		x			x		x				x				x		x			x
Errichetti, Gimpel, Boulet	2002	x														x	x	x						x		x		x		x			x		x				x				x		x			x
Frazer, Miller	1977	x							x								x						x			x	organ system		>3-4 weeks per system		x				x	x					x				x			x
Gall, Meredith, Stillman, Rutala, Gooden, Boyer, Riggs	1984	x		x		x																x	x			x	musculoskeletal		30 hours				x	$5-7/hour					x				x	5		x		
Gruppen, Branch, Laing	1996	x							x									x					x		120 min		musculoskeletal		50 hours in-person and 50		x				x				x				x	20		x		
Haq, Fuller, Dacre	2006	x														x			x				x		2x75 min		musculoskeletal, arthritis		2 full-day and 4 half-day				x	£10 honorarium and £5 travel				x		x				3		x		
Hasle, Anderson, Szerlip	1994	x					x											x					x		60 min		abdomen, cardiovascular, head and neck, musculoskeletal, neurology, pulmonary, vital signs			x			x	$75/hour training, $20/hour		x							x	7-10				x
Hendry, Schrieber, Bryce	1999	x											X							x				x	80 min		musculoskeletal, arthritis		3 days		x				x	x							x	4		x		
Hoefer, Sterz, Bender, Stefanescu, Theis, Walcher, Sader, Ruesseler	2017	x														x			x				x		60 min		complex head and skull		240 min				x	€10/hour		x					x			2			x	
Howley, Gliva-McConvey, Thornton	2009				x											x						x		x		x		x		x			x	$17/hour (mean and median)					x				x		x			x
Humphrey-Murto, Smith, Touchie, Wood	2004	x									x							x					x		3x180 min		musculoskeletal			x			x		x				x				x		x	x		
Laguna, Stillman	1978	x														x						x	x			x	neurological		20 hours		x				x				x				x	2				x

Implementation and Utilization of Physical Examination Teaching Associate (PETA) Programs: A Scoping Review

Online Supplementary Materials

Table 3. PETA Program Structure and Utilization Patterns

Author(s)	Year of Publication	Learner type				Number of learners in each session											Timing of initial PETA instructional session within curriculum						How is learner preparation prior to instructional session with PETA described?		Session length		Type of physical examination(s) instructed		Length of initial training for PETA		Were PETAs independent or paired for initial instructional session(s)?			Wage		Prior to PETA involvement, who instructed the students?				Prior to PETA involvement, who/what received the exams?				Number of PETAs in program		Were PETAs included because of pre-existing pathology?		
		Medical Student(s)	Nurse Practitioner Student(s)	Physician(s)	Not Addressed	1	2	3	4	5	6	7	8	9	10 or more	Not Addressed	1st year	2nd year	3rd year	4th year	5th year	Not Addressed	Description Provided	Not Addressed	Time Provided	Not Addressed	Specific Examination(s)	Not Specified	Time Provided	Not Addressed	Independent	Paired with Peer (PETA)	Not Addressed	Wage Provided	Not Addressed	Faculty	Senior Medical Students	Other	Not Addressed	Clinic Patients	Peers	SP	Not Addressed	Number Provided	Not Addressed	Yes	No	Not Addressed
Martineau, Mamede, St-Onge, Rikers, Schmidt	2013	x				x												x					x		45 min (1 learner), 135 min (3 learners)		head-to-toe		30 hours		x				x	x				x		x			x			x
Oswald, Bell, Wiseman, Snell	2011	x							x									x					x		120 min		musculoskeletal, major joints		100 hours		x				x	x							x		x	x		
Oswald, Wiseman, Bell, Snell	2011	x							x									x					x		120 min		musculoskeletal			x	x				x	x							X		x	x		
Parle, Ross, Coffey	2012				x											x						x		x		x				x			x		x				x				X		x			x
Raj, Badcock, Brown, Deighton, O'Reilly	2006	x													x						x			x	2x60 min		hand or knee		>23 hours		x	x		~£10/half day,~£20/full day, plus travel				x					X	11		x		
Riggs, Gall, Meredith, Boyer, Gooden	1982	x		x												x						x		x		x	musculoskeletal			x			x		x				x				X	5		x		
Sachdeva, Wolfson, Blair, Gillum, Gracely, Friedman	1997	x				x													x				x		40 min		abdominal			x					x	x				X					x			x
Schrieber, Hendry, Hunter	2000	x						x									x							x	80 min		hand, wrist			x			x		x				x				X		x	x		
Smith, Henry-Edwards, Shanahan, Ahern	2000	x														x		x						x		x	musculoskeletal			x			x		x				x	X					x	x		
Stillman	1984	x		x												Other	x	x	x				x			x	complete exam, cardiovascular, musculoskeletal, orthopedic, neurological, pulmonary		15 hours				x		x				x				X		x	x		
Stillman, Levinson, Ruggill, Sabers	1979		x													x						x		x		x		x	25 hours				x		x				x	X				2			x	
Stillman, Ruggill, Rutala, Sabers	1980	x				x												x	x				x			x	cardiovascular, pulmonary		>25 hours		x			$6/hour					x				X	7		x		
Stillman, Ruggill, Rutala, Sabers	1979	x				x																x	x			x	cardiovacscular, pulmonary		25 hours		x				x				x				X	7		x		
Wykurz, Kelly	2002				x											x						x		x		x	musculoskeletal		Variable				x		x				x				X		x	x	x	
Zabel, Sterz, Hoefer, Stefanescu, Lehmann, Sakmen, Marzi, Ruesseler	2019	x										x							x				x		50 min		knee, shoulder		3 hours				x	€15/hour					x				X	6			x	
