# Supplementary material for: Implementation and utilization of Physical Examination Teaching Associate (PETA) programs: a scoping review
Source: Adv Simul (Lond). 2026 Feb 11;11:13. doi: 10.1186/s41077-026-00416-z (PMC12922200; doi:10.1186/s41077-026-00416-z)
Supplement: Supplementary file 4 — Supplementary Material 4. Table 4. Implementation of PETA Programs. Table of whether details were provided about the ASPE SOBP Domains (safe work environment, instructional session development, PETA training, program management, professional development). [file 41077_2026_416_MOESM4_ESM.pdf]

Implementation and Utilization of Physical Examination Teaching Associate (PETA) Programs: A Scoping Review

Online Supplementary Materials

Table 4. Implementation of PETA Programs

Author(s)	Year of Publication	Were details provided about Safe Work Environment?			Were details provided about Instructional Session Development?			Were details provided about PETA training?			Were details provided about Program Management?			Were details provided about Professional Development?		
		Yes	No	Not Applicable	Yes	No	Not Applicable	Yes	No	Not Applicable	Yes	No	Not Applicable	Yes	No	Not Applicable
Aamodt, Virtue, Dobbie	2006		x		x			x				x			x	
Allen, Miller, Ratner, Santilli	2011		x		x			x			x				x	
Barley, Fisher, Dwinnell, White	2006		x		x			x			x				x	
Barnes, Albanese, Schroeder, Reiter	1978		x			x		x				x			x	
Bell, Badley, Glazier, Poldre	1997		x			x		x				x			x	
Branch, Graves, Hanczyc, Lipsky	1999		x		x			x				x			x	
Branch, Lipsky	1998		x			x		x				x			x	
Danielson, Venugopal, Mefford, Clarke	2019			x			x			x			x			x
Errichetti, Gimpel, Boulet	2002			x			x			x	x					x
Frazer, Miller	1977		x		x			x				x			x	
Gall, Meredith, Stillman, Rutala, Gooden, Boyer, Riggs	1984	x			x			x			x				x	
Gruppen, Branch, Laing	1996		x		x			x			x				x	
Haq, Fuller, Dacre	2006	x			x			x			x				x	

Implementation and Utilization of Physical Examination Teaching Associate (PETA) Programs: A Scoping Review

Online Supplementary Materials

Table 4. Implementation of PETA Programs

Author(s)	Year of Publication	Were details provided about Safe Work Environment?			Were details provided about Instructional Session Development?			Were details provided about PETA training?			Were details provided about Program Management?			Were details provided about Professional Development?		
		Yes	No	Not Applicable	Yes	No	Not Applicable	Yes	No	Not Applicable	Yes	No	Not Applicable	Yes	No	Not Applicable
Hasle, Anderson, Szerlip	1994		x		x			x			x				x	
Hendry, Schrieber, Bryce	1999		x			x		x			x				x	
Hoefer, Sterz, Bender, Stefanescu, Theis, Walcher, Sader, Ruesseler	2017		x		x			x				x			x	
Howley, Gliva-McConvey, Thornton	2009			x			x			x			x			x
Humphrey-Murto, Smith, Touchie, Wood	2004		x			x		x				x			x	
Laguna, Stillman	1978		x			x		x				x			x	
Martineau, Mamede, St-Onge, Rikers, Schmidt	2013		x		x			x				x			x	
Oswald, Bell, Wiseman, Snell	2011		x		x			x				x			x	
Oswald, Wiseman, Bell, Snell	2011		x		x				x			x			x	
Parle, Ross, Coffey	2012		x			x			x				x	x		
Raj, Badcock, Brown, Deighton, O'Reilly	2006			x	x			x				x			x	
Riggs, Gall, Meredith, Boyer, Gooden	1982	x				x		x				x			x	
Sachdeva, Wolfson, Blair, Gillum, Gracely, Friedman	1997		x		x			x				x			x	

Implementation and Utilization of Physical Examination Teaching Associate (PETA) Programs: A Scoping Review

Online Supplementary Materials

Table 4. Implementation of PETA Programs

Author(s)	Year of Publication	Were details provided about Safe Work Environment?			Were details provided about Instructional Session Development?			Were details provided about PETA training?			Were details provided about Program Management?			Were details provided about Professional Development?		
		Yes	No	Not Applicable	Yes	No	Not Applicable	Yes	No	Not Applicable	Yes	No	Not Applicable	Yes	No	Not Applicable
Schrieber, Hendry, Hunter	2000		x			x		x				x			x	
Smith, Henry-Edwards, Shanahan, Ahern	2000		x			x			x			x			x	
Stillman	1984		x		x			x			x			x		
Stillman, Levinson, Ruggill, Sabers	1979		x				x	x				x			x	
Stillman, Ruggill, Rutala, Sabers	1980		x		x			x			x				x	
Stillman, Ruggill, Rutala, Sabers	1979	x			x			x			x				x	
Wykurz, Kelly	2002	x					x	x					x			x
Zabel, Sterz, Hoefer, Stefanescu, Lehmann, Sakmen, Marzi, Ruesseler	2019		x		x			x				x			x	
