# Supplementary material for: Implementation and utilization of Physical Examination Teaching Associate (PETA) programs: a scoping review
Source: Adv Simul (Lond). 2026 Feb 11;11:13. doi: 10.1186/s41077-026-00416-z (PMC12922200; doi:10.1186/s41077-026-00416-z)
Supplement: Supplementary file 5 — Supplementary Material 5. Studies meeting inclusion criteria. [file 41077_2026_416_MOESM5_ESM.pdf]

**Implementation and Utilization of Physical Examination Teaching Associate (PETA) Programs: A Scoping Review**
**Online Supplementary Materials**
**Studies Meeting Inclusion Criteria**


1. Aamodt CB, Virtue DW, Dobbie AE. Trained standardized patients can train their peers to provide well-rated, cost-effective physical exam skills training to first-year medical students. Fam Med. 2006 May;38(5):326–9.
2. Allen SS, Miller J, Ratner E, Santilli J. The educational and financial impact of using patient educators to teach introductory physical exam skills. Med Teach. 2011;33(11):911–8.
3. Barley GE, Fisher J, Dwinnell B, White K. Teaching foundational physical examination skills: study results comparing lay teaching associates and physician instructors. Acad Med. 2006 Oct;81(10 Suppl):S95-97.
4. Barnes HV, Albanese M, Schroeder J, Reiter S. Senior medical students teaching the basic skills of history and physical examination. J Med Educ. 1978 May;53(5):432–4.
5. Bell MJ, Badley EM, Glazier RH, Poldre P. A pilot study to determine the effect of patient educators on medical students' and residents' skills in joint examination. Acad Med. 1997 Oct;72(10):919.
6. Branch VK, Graves G, Hanczyc M, Lipsky PE. The utility of trained arthritis patient educators in the evaluation and improvement of musculoskeletal examination skills of physicians in training. Arthritis Care Res. 1999 Feb;12(1):61–9.
7. Branch VK, Lipsky PE. Positive impact of an intervention by arthritis educators on retention of information, confidence, and examination skills of medical students. Arthritis Care Res. 1998 Feb;11(1):32–8.
8. Danielson AR, Venugopal S, Mefford JM, Clarke SO. How do novices learn physical examination skills? A systematic review of the literature. Med Educ Online. 2019 Dec;24(1):1608142.
9. Errichetti AM, Gimpel JR, Boulet JR. State of the art in standardized patient programs: a survey of osteopathic medical schools. J Am Osteopath Assoc. 2002 Nov;102(11):627–31.
10. Frazer NB, Miller RH. Training Practical Instructors (Programmed Patients) to Teach Basic Physical Examination. Journal of Medical Education. 52:149–51.
11. Gall EP, Meredith KE, Stillman PL, Rutala PJ, Gooden MA, Boyer JT, et al. The use of trained patient instructors for teaching and assessing rheumatologic care. Arthritis Rheum. 1984 May;27(5):557–63.
12. Gruppen LD, Branch VK, Laing TJ. The use of trained patient educators with rheumatoid arthritis to teach medical students. Arthritis Care Res. 1996 Aug;9(4):302–8.

13. Haq I, Fuller J, Dacre J. The use of patient partners with back pain to teach undergraduate medical students. Rheumatology (Oxford). 2006 Apr;45(4):430–4.
14. Hasle JL, Anderson DS, Szerlip HM. Analysis of the costs and benefits of using standardized patients to help teach physical diagnosis. Acad Med. 1994 Jul;69(7):567–70.
15. Hendry GD, Schrieber L, Bryce D. Patients teach students: partners in arthritis education. Med Educ. 1999 Sep;33(9):674–7.
16. Hoefer SH, Sterz J, Bender B, Stefanescu MC, Theis M, Walcher F, et al. Conveying practical clinical skills with the help of teaching associates-a randomised trial with focus on the long term learning retention. BMC Med Educ. 2017 Mar 28;17(1):65.
17. Howley LD, Gliva-McCorvey G, Thorton J. Standardized Patient Practices: Initial Report on the Survey of US and Canadian Medical Schools. MEO [Internet]. 2009 Jun 29 [cited 2024 Aug 27];14. Available from: http://med-ed-online.net/index.php/meo/article/view/4513
18. Humphrey-Murto S, Smith CD, Touchie C, Wood TC. Teaching the musculoskeletal examination: are patient educators as effective as rheumatology faculty? Teach Learn Med. 2004;16(2):175–80.
19. Laguna JF, Stillman PL. Teaching undergraduate medical students the neurological examination. J Med Educ. 1978 Dec;53(12):990–2.
20. Martineau B, Mamede S, St-Onge C, Rikers RMJP, Schmidt HG. To observe or not to observe peers when learning physical examination skills; that is the question. BMC Med Educ. 2013 Apr 17;13:55.
21. Oswald AE, Bell MJ, Wiseman J, Snell L. The impact of trained patient educators on musculoskeletal clinical skills attainment in pre-clerkship medical students. BMC Med Educ. 2011 Sep 23;11:65.
22. Oswald AE, Wiseman J, Bell MJ, Snell L. Musculoskeletal examination teaching by patients versus physicians: how are they different? Neither better nor worse, but complementary. Med Teach. 2011;33(5):e227-235.
23. Parle J, Ross N, Coffey F. Clinical teaching associates in medical education: the benefits of certification. Clin Teach. 2012 Oct;9(5):275–9.
24. Raj N, Badcock LJ, Brown GA, Deighton CM, O'Reilly SC. Undergraduate musculoskeletal examination teaching by trained patient educators--a comparison with doctor-led teaching. Rheumatology (Oxford). 2006 Nov;45(11):1404–8.
25. Riggs GE, Gall EP, Meredith KE, Boyer JT, Gooden A. Impact of intensive education and interaction with health professionals on patient instructors. J Med Educ. 1982 Jul;57(7):550–6.

26. Sachdeva AK, Wolfson PJ, Blair PG, Gillum DR, Gracely EJ, Friedman M. Impact of a standardized patient intervention to teach breast and abdominal examination skills to third-year medical students at two institutions. Am J Surg. 1997 Apr;173(4):320–5.
27. Schrieber L, Hendry GD, Hunter D. Musculoskeletal examination teaching in rheumatoid arthritis education: trained patient educators compared to nonspecialist doctors. J Rheumatol. 2000 Jun;27(6):1531–2.
28. Smith MD, Henry-Edwards S, Shanahan EM, Ahern MJ. Evaluation of patient partners in the teaching of the musculoskeletal examination. J Rheumatol. 2000 Jun;27(6):1533–7.
29. Stillman PL. Expanding the role of nonphysician teachers and evaluators. J Am Med Womens Assoc (1972). 1984;39(2):54–6.
30. Stillman PL, Levinson D, Ruggill J, Sabers D. An objective method of assessing physical examination skills of nurse practitioners. J Nurs Educ. 1979 Mar;18(3):31–2.
31. Stillman PL, Ruggill JS, Rutala PJ, Sabers DL. Patient instructors as teachers and evaluators. J Med Educ. 1980 Mar;55(3):186–93.
32. Stillman P, Ruggill J, Rutala P, Sabers D. An instructional program using patient instructors as teachers and evaluators. Annu Conf Res Med Educ. 1979 Nov;18:53–8.
33. Wykurz G, Kelly D. Developing the role of patients as teachers: literature review. BMJ. 2002 Oct 12;325(7368):818–21.
34. Zabel J, Sterz J, Hoefer SH, Stefanescu MC, Lehmann M, Sakmen DK, et al. The Use of Teaching Associates for Knee and Shoulder Examination: A Comparative Effectiveness Analysis. J Surg Educ. 2019;76(5):1440–9.
